# Exploring the clinical outcome of Mindfulness-Based Cognitive Therapy for bipolar and unipolar depressive patients in routine clinical practice: a pilot study

**DOI:** 10.1186/s40345-019-0153-0

**Published:** 2019-08-28

**Authors:** I. Hanssen, M. I. van Dord, F. R. Compen, D. E. M. Geurts, M. P. J. Schellekens, A. E. M. Speckens

**Affiliations:** 1 Department of Psychiatry, Centre for Mindfulness, Radboud University Medical Centre, Postbus 9101, 6500 HB Nijmegen, The Netherlands; 2 Donders Institute for Brain, Cognition and Behaviour, Radboud University, Nijmegen, The Netherlands; 3 Centre for Psycho-Oncology, Helen Dowling Institute, Bilthoven, The Netherlands

**Keywords:** Mindfulness Based Cognitive Therapy, Bipolar disorder, Unipolar depressive disorder, Feasibility, Effectiveness

## Abstract

**Background:**

Mindfulness Based Cognitive Therapy (MBCT) has been adopted as an evidence-based treatment for unipolar depressive disorder (UDD). Although MBCT has not been included in the treatment guidelines for bipolar disorder (BD), MBCT is regularly being offered to patients with BD in routine clinical practice. In this pilot study we used routine outcome monitoring (ROM) data to explore whether there are indications that patients with BD might benefit less from MBCT than patients with UDD in terms of feasibility and effectiveness.

**Methods:**

The study population consisted of patients with BD (*n* = 30) or UDD (*n* = 501) who received MBCT at the Radboudumc Centre for Mindfulness in Nijmegen, the Netherlands. Patients completed self-report measures of depressive symptom severity, worry, well-being, mindfulness skills and self-compassion pre- and post MBCT as part of the ROM.

**Results:**

There were significant less patients with BD who decided to start MBCT after intake than patients with UDD. No differences in dropout between groups were found. Results showed significant moderate to large improvements in both groups after MBCT, while no differences between groups were found, on all outcome measures.

**Conclusions:**

This study demonstrates that there are no indications that MBCT, when delivered in heterogeneous patient groups in routine clinical practice, is less beneficial for patients with BD than patients with UDD in terms of feasibility and effectiveness. This lends support to conduct an adequately powered RCT to examine the (cost-)effectiveness of MBCT in BD as the next step before implementing MBCT on a larger scale in patients with BD.

## Background

Bipolar disorder (BD) is a severe, chronic condition that belongs to the leading causes of disability worldwide (Ferrari et al. 2016). Despite multiple available evidence-based pharmacological and psychological interventions for bipolar disorder, treatment outcomes are modest at best, as a result of which patients with bipolar disorder often do not achieve full remission and experience substantial residual mood symptoms between episodes (Chisholm et al. 2005; Judd et al. 2003). Augmenting, evidence-based psychotherapies are necessary in order to improve treatment outcome for patients with bipolar disorder. Lately, there is an increasing interest in the potential of mindfulness-based approaches to improve outcomes of patients with several psychiatric conditions (Chu et al. 2018).

Mindfulness-Based Cognitive Therapy (MBCT) integrates elements of cognitive therapy with meditative practices. Patients develop the capacity to become aware of distressing thoughts, emotions, and bodily sensations in a non-judgmental way, which helps them to disengage from dysfunctional habit patterns (Segal et al. 2012). MBCT was originally developed to prevent depressive relapse in unipolar depressive disorder (UDD) and has been shown effective in reducing relapse (e.g. Kuyken et al. 2016), as well as in reducing subsyndromal and acute depressive symptoms (Kingston et al. 2007; van Aalderen et al. 2012). Therefore, the National Institute of Clinical Excellence (NICE) (2018) recommends MBCT as a relapse prevention approach for unipolar depressive patients with a history of three or more depressive episodes.

Although MBCT has not yet been included as a treatment option in the NICE-guidelines for bipolar disorder, it is regularly being offered to patients with bipolar disorder in routine clinical practice (Bos et al. 2014). Several previous studies have investigated the clinical effectiveness of MBCT in bipolar disorder (Deckersbach et al. 2012; Miklowitz et al. 2009; Williams et al. 2008; Perich et al. 2013; Weber et al. 2010, 2017), and, despite limited sample sizes, results seem to support the possible application of MBCT in patients with bipolar disorder. A recent systematic literature review suggested a positive effect of MBCT on residual depression, anxiety, and mood regulation, while it did not appear to precipitate manic symptoms (Lovas and Schuman-Olivier 2018). However, the feasibility and effectiveness of MBCT for bipolar disorder was only studied in small, underpowered studies (Lovas and Schuman-Olivier 2018).

In order to address this issue, the present pilot study examined the feasibility and effectiveness of MBCT in patients with bipolar disorder as compared to those with unipolar depressive disorder in a naturalistic cohort in routine clinical practice. Our aim was to explore whether there are any indications that MBCT for bipolar disorder might result in a higher dropout or a reduced effectiveness in terms of depressive symptom severity, worry, well-being, mindfulness skills, and self-compassion compared to patients with unipolar depressive disorder.

## Methods

### Participants

The study population consisted of a naturalistic cohort of patients with either bipolar or unipolar depressive disorder (18+ years old) as assessed with the Mini International Neuropsychiatric Interview Plus (M.I.N.I Plus) (Sheehan et al. 1998). Patients received MBCT at the Radboudumc Centre for Mindfulness in Nijmegen, the Netherlands, between July 2012 and June 2016. Patients were referred to the Centre for Mindfulness by either their general practitioner or their attending psychologist or psychiatrist.

### Procedure

During a diagnostic clinical assessment by means of the M.I.N.I Plus (Sheehan et al. 1998), it was checked whether MBCT seemed suitable, or whether other evidence-based treatments were deemed more appropriate, such as cognitive behavioral therapy or trauma therapy. Patients were invited to participate in MBCT when they were willing to participate in a group setting, to adhere to homework assignments, and to be able to attend at least six out of eight sessions. In the Netherlands, Routine Outcome Monitoring (ROM) of generic and disorder-specific outcomes is part of general clinical practice (De Beurs et al. 2011). As part of the ROM, patients had to complete a set of measures (see below) at least 2 weeks prior to the start of MBCT. After the final MBCT-session, patients were asked to complete the measures on site for a second time. Therefore, there were 11 weeks between pre- and post-assessment in total. We received approval from the ethical committee of Radboudumc to use these anonymized data for research purposes (CWOnr = 2015 1972).

### Measures

Self-report measures were used to assess depressive symptom severity, worry, well-being, mindfulness skills and self-compassion. Depressive symptom severity was measured with the 21-item Beck Depression Inventory (BDI-II) (Beck et al. 1996). Worry was measured with the 16-item Penn State Worry Questionnaire (PSWQ) (Meyer et al. 1990; van Rijsoort et al. 1999). Well-being in terms of symptom distress, interpersonal relations and social role performance were measured with the 45-item Outcome Questionnaire (OQ-45) (de Jong et al. 2007; Lambert et al. 1996). Mindfulness skills were measured with the 24-item Five Facet Mindfulness Questionnaire Short Form (FFMQ-SF) (Bohlmeijer et al. 2011). Self-compassion was measured with the 12-item Self-Compassion Scale-Short Form (SCS-SF) (Raes et al. 2011). The psychometric properties of all instruments are considered to be adequate to good.

### Intervention

In accordance with the MBCT-program originally developed by Segal, Williams and Teasdale (Segal et al. 2002), the group-based MBCT consisted of eight weekly 2.5 h sessions, a silent day between session six and seven, and home assignments for 30–45 min per day. Each MBCT-group consisted of 8 to 12 participants. MBCT was being taught to heterogeneous patient groups, mostly consisting of patients with unipolar depressive disorder, either currently depressed or in (partial) remission, but also including patients with anxiety disorder, ADHD, autism or bipolar disorder. During the sessions, participants were offered guided meditation exercises, psycho-education, and dialogue about the exercises. The MBCT were taught by qualified teachers meeting the advanced criteria of the Association of Mindfulness Based Teachers in the Netherlands and Flanders, which are in concordance with the good practice guidelines of the UK Network for Mindfulness-Based Teachers (2010). The competency of the teachers had been evaluated between ‘advanced beginner’ and ‘competent’ by means of the MBI-TAC (Crane et al. 2013) in earlier randomized controlled studies conducted at the Centre for Mindfulness (e.g. Huijbers et al. 2015). The mean teacher competency score was 3.4 (SD = 0.7, range 2.2–4.3) on a Likert-scale from 1 to 6.

### Data analysis

Data were analyzed using the Statistical Package for the Social Sciences (SPSS) version 22.0 (2013) on an intention to treat basis. Descriptive statistics of clinical and demographic variables of patients with bipolar or unipolar depressive disorder were calculated and compared with χ-square or ANOVA statistics. Separate Linear Mixed-Effects Models were used to test whether scores on each outcome improved from pre- to post-treatment (within-group), and whether there were any indications of treatment outcome being less favorable in patients with bipolar versus unipolar depressive disorder (between group). Time, group and their interaction were added as fixed effects, and a random intercept for participants was included (to reflect differences between participants). Diagonal covariance structures were used. In addition, a sensitivity analysis was conducted including both random intercepts and random slopes for factor time. Furthermore, effect sizes were calculated. Within-group effect sizes *d* were calculated by dividing the differences in outcomes over time by the standard deviation in the relevant group at baseline. Between-group effect sizes *d* were calculated by dividing the differences in outcomes between groups at post-intervention by the pooled standard deviation at baseline. Effect sizes of 0.20, 0.50 and 0.80 were considered small, medium, and large, respectively (Cohen 1992).

## Results

### Demographics

Demographic and clinical characteristics of both patients with bipolar and unipolar depressive disorder are shown in Table 1. Overall, patients were often female (65.5%) and highly educated (55.6%). Groups did not differ in terms of gender, level of education, work status, or age. In terms of marital status, patients with bipolar disorder were significantly more often divorced, separated, or widowed and patients with unipolar depressive disorder were more often single. In terms of clinical characteristics, there were no differences between patients with bipolar and unipolar depressive disorder in terms of being currently depressed or having previously received cognitive behavioral therapy. Patients with bipolar disorder more often used mood stabilizers and antipsychotics, whilst patients with unipolar depressive disorder more often used antidepressants. In patients with bipolar and unipolar disorder, 14 (46.7%) and 218 (43.5%) patients respectively suffered from co-morbid psychiatric disorders, such as generalized anxiety disorder, panic disorder, post-traumatic stress disorder, and substance abuse (current or in past).

**Table 1:** Demographic and clinical characteristics

	BD (*n* = 30) Mean (SD)	MDD (*n* = 501) Mean (SD)	*t*	*p*
Age	49.4 (12.6)	46.9 (13.0)	− 1.467	0.143

	*n* (%)	*n* (%)	Chi square	*p*
***Gender***			1.26	0.262
Female	23 (76.7)	325 (64.9)		
Male	7 (23.3)	176 (35.1)		
***Marital status***			*7.18*	*0.028*
Single	4 (13.3)	123 (26.1)		
Married/living with a partner	16 (53.3)	275 (58.3)		
Divorced/separated/widowed	10 (33.3)	74 (15.7)		
***Education*** ^a^			1.07	0.586
Low	1 (4.3)	35 (9.3)		
Intermediate	3 (13.0)	65 (17.3)		
High	19 (82.6)	276 (73.4)		
***Work***			5.65	0.227
Working or student	12 (41.4)	245 (53.5)		
Unemployed	5 (17.2)	78 (17.0)		
Retired	2 (6.9)	42 (9.2)		
Unemployed due to illness	3 (10.3)	46 (10.0)		
Incapacitated	7 (24.1)	47 (10.3)		
***Current depression***			0.32	0.572
Yes	15 (50)	224 (44.7)		
No	15 (50)	277 (55.3)		
***Current pharmacological treatment***			*4.80*	*0.029*
Antidepressants	10 (33.3)	240 (47.9)		
Mood stabilizers	19 (63.3)	1 (0.2)		
Antipsychotics	10 (33.3)	40 (8.0)		
Benzodiazepines	5 (17.2)	81 (16.6)		
***Previous non-pharmacological treatment***			1.34	0.248
Cognitive (Behavioral) therapy	11 (37.9)	238 (49.0)		
***DSM-IV co-morbid diagnosis***				
Generalized anxiety disorder	2 (6.7)	36 (7.2)		
Panic Disorder				
With agoraphobia	1 (3.3)	22 (4.4)		
Without agoraphobia	2 (6.7)	23 (4.6)		
Post-traumatic stress disorder	3 (10.0)	16 (3.2)		
Social anxiety disorder	0 (0.0)	30 (6.0)		
Obsessive compulsive disorder	0 (0.0)	15 (3.0)		
Alcohol abuse				
Current	1 (3.3)	13 (2.6)		
In past	4 (13.3)	19 (3.8)		
Autism spectrum disorder	1 (3.3)	10 (2.0)		
Attention deficit disorder	0 (0.0)	34 (6.8)		

Italic numbers indicate significant difference

^a^Education level: low = elementary school/preparatory vocational education; intermediate = vocational education; high = pre-university high school/community college/university

### Feasibility and acceptance

**Fig. 1:**
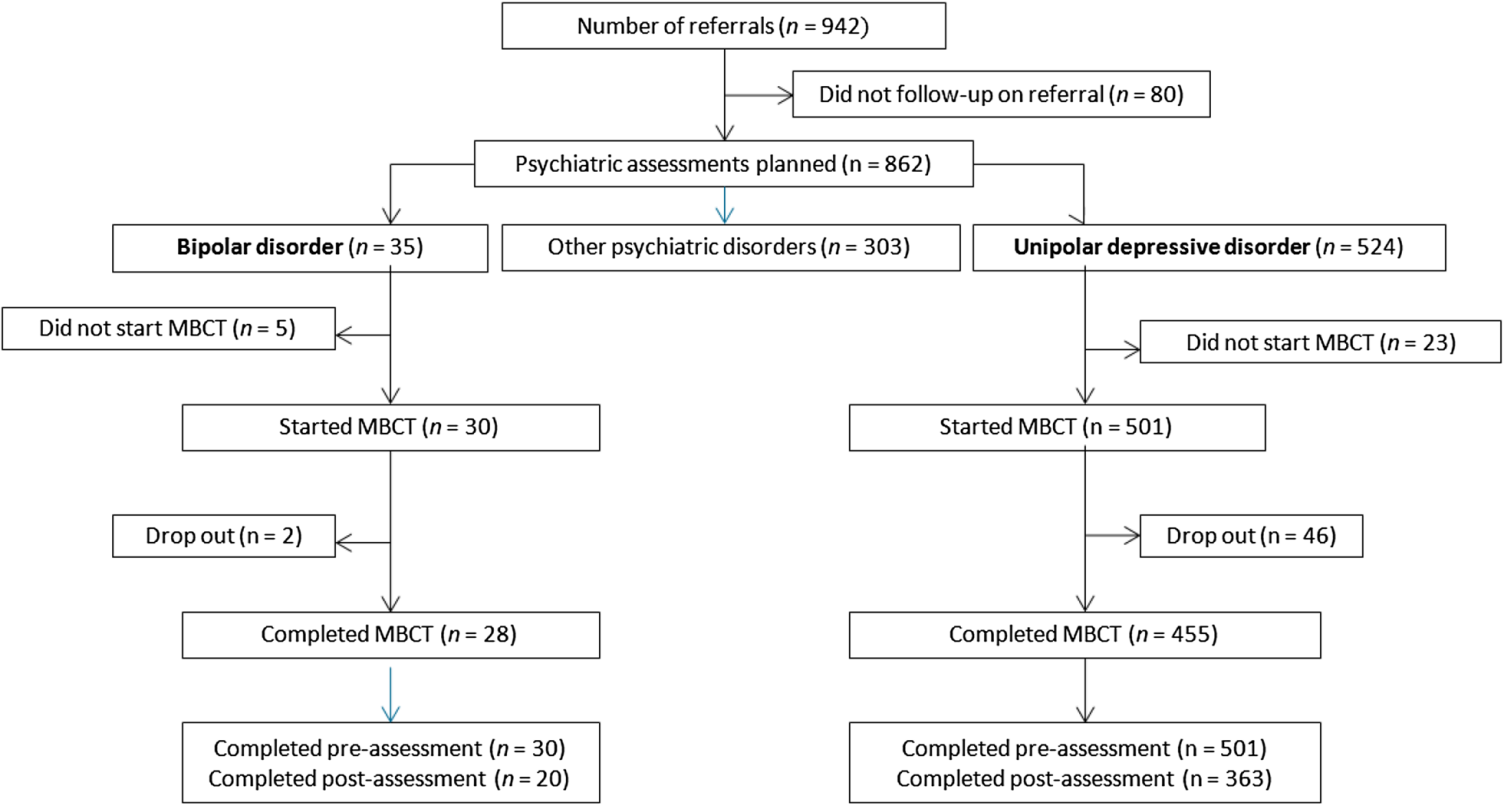
Flowchart of patients with BD and UDD

See Fig. 1 for a flowchart of the study population. During the study period a total of 942 patients were referred for MBCT, of whom 862 (91.5%) completed the psychiatric assessment. Of these patients, 35 (4.1%) were diagnosed with bipolar and 524 (60.8%) with unipolar depressive disorder. Eventually, 30 (85.7%) patients with bipolar disorder (19 bipolar type I and 11 bipolar type II) and 501 (95.6%) with unipolar depressive disorder started with MBCT. This difference was significant (*η* = 6.753, *p* = 0.009). The reasons for patients with bipolar disorder to withdraw before the start of MBCT were: advice to start other evidence-based treatments first, such as psycho-education and trauma therapy (*n* = 4), and hospital admission due to a severe depressive episode (*n* = 1). The proportion of patients who started MBCT and completed four or more sessions was very similar in both groups, 28 (93.3%) in patients with bipolar disorder and 455 (90.8%) of those with unipolar depressive disorder. Reasons of the two patients with bipolar disorder to drop out of MBCT were lack of time and starting an alternative psychological treatment. Furthermore, two patients with bipolar disorder did not attend the last MBCT-session due to severe depressive symptoms and suicidality. The two groups did not differ either in mean number of sessions patients attended: 7.27 (SD = 2.05) in patients with bipolar disorder and 7.88 (SD = 1.80) in those with unipolar depressive disorder.

### Effectiveness of MBCT

Of the 531 patients who started MBCT, 383 (72.1%) completed the post-treatment measures, 20 (66.7%) in the BD and 363 (72.9%) in the MDD group (*η* = 0.612, *p* = 0.434). The identified reasons for the ten bipolar patients not to complete post-assessments were as follows; drop out (2), attended last MBCT-session but could not fill out the assessment on site and did not send back per post (3), or did not attend last MBCT-session due to suicidality (1), severe depression (1), vacation (1), or unknown reasons (2).

The effectiveness of MBCT in both patients with bipolar and unipolar depressive disorder is shown in Table 2. In both groups, MBCT resulted in a significant reduction of depressive symptoms with a moderate effect size. Patients with bipolar disorder reported a large reduction of worry and those with unipolar depressive disorder a moderate reduction. Both groups reported significant improvements of overall well-being, mindfulness and self-compassion following MBCT, with moderate effect sizes.

**Table 2:** Effect of intervention within and between groups

	Within BD group				Within MDD group				Between groups		
	BD (*n* = 20, 67%) Mean (SD)	*F* (df)	*p*	*d*	MDD (*n* = 365, 73%) Mean (SD)	*F* (df)	*p*	*d*	*F* (df)	*p*	*d*
***BDI-II***		*10.28* (1.24)	*0.004*	− 0.72		*287.84* (1.391)	*<* 0*.001*	− 0.76	0.11 (1.432)	0.743	0.00
Pre	21.8 (11.9)				21.6 (10.5)						
Post	13.2 (10.9)				13.6 (10.0)						
***PSWQ***		*19.43* (1.20)	*<* *0.001*	− 0.90		*172.57* (1.381)	*<* 0*.001*	− 0.52	3.87 (1.416)	0.05	− 0.02
Pre	60.1 (11.8)				59.4 (12.3)						
Post	49.5 (11.7)				53.0 (12.2)						
***OQ***											
***Symptom distress***		*6.51* (1.14)	*0.023*	− 0.52		*185.83* (1.337)	*<* *0.001*	− 0.63	0.268 (1.381)	0.614	0.00
Pre	45.7 (12.8)				47.2 (12.9)						
Post	39.0 (16.5)				39.1 (13.3)						
***Interpersonal relations***		2.57 (1.15)	0.130	− 0.25		*56.66* (1.335)	*<* 0*.001*	− 0.31	0.04 (1.368)	0.841	− 0.05
Pre	14.3 (6.7)				16.2 (6.1)						
Post	12.6 (6.3)				14.3 (6.0)						
***Social role performance***		3.26 (1.16)	0.090	− 0.49		*71.74* (1.334)	*<* 0*.001*	− 0.41	0.01 (1.381)	0.910	0.02
Pre	14.4 (3.9)				13.9 (4.6)						
Post	12.5 (4.4)				12.0 (4.8)						
***FFMQ***		*9.76* (1.25)	*0.005*	0.63		*180.77* (1.407)	*<* 0*.001*	0.61	0.46 (1.434)	0.516	0.01
Pre	71.2 (14.0)				70.9 (11.6)						
Post	80.0 (13.5)				78.0 (11.0)						
***Observing***		4.11 (1.21)	0.055	0.32		*104.68* (1.415)	*<* 0*.001*	0.44	0.03 (1.430)	0.726	0.04
Pre	14.3 (3.7)				13.7 (3.2)						
Post	15.5 (3.4)				15.1 (2.8)						
*** Describing***		1.54 (1.22)	0.228	0.16		*19.07* (1.400)	*<* 0*.001*	0.17	0.06 (1.417)	0.796	0.00
Pre	16.6 (5.6)				16.8 (4.2)						
Post	17.5 (5.0)	4.05 (1.20)	0.058	0.52	17.5 (3.7)	*62.97* (1.108)	*<* 0*.001*	0.35	0.12 (1.440)	0.791	0.00
***Acting with awareness***											
Pre	13.7 (3.1)				14.0 (3.7)						
Post	15.3 (3.2)				15.3 (3.2)						
*** Non-judging***		*7.80* (1.19)	*0.012*	0.63		*94.61* (1.405)	*<* 0*.001*	0.45	0.89 (1.440)	0.358	0.06
Pre	13.9 (4.0)				13.8 (3.8)						
Post	16.4 (3.9)				15.5 (3.7)						
*** Non-reactivity***		*15.17* (1.21)	*0.001*	0.82		*190.60* (1.394)	*<* 0*.001*	0.64	0.91 (1.430)	0.341	0.08
Pre	12.7 (3.4)				12.5 (3.3)						
Post	15.5 (3.5)				14.6 (3.1)						
***SCS***		*11.39* (1.18)	*0.003*	0.67		*263.13* (1.386)	*<* 0*.001*	0.76	0.01 (1.416)	0.925	0.00
Pre	21.2 (4.3)				21.3 (3.7)						
Post	24.1 (3.7)				24.1 (3.7)						

Italic numbers indicate significant difference

Positive *d*’s indicate increases in scores over time and negative *d*’s indicate decreases in scores over time

Reduction of depressive symptoms and improvements of well-being, mindfulness, and self-compassion did not differ between groups. This implies that there was no indication of a lower effectiveness of MBCT in patients with bipolar disorder versus those with unipolar depressive disorder, as evidenced by the absence of significant time by intervention interaction effects (see Table 2). Reductions of worry, however, tended to be larger in bipolar compared to unipolar patients.

Sensitivity analyses of the primary outcome (depressive symptoms measures with BDI) including random slopes for factor time showed similar results.

## Discussion

This pilot study investigated feasibility and effectiveness of MBCT in patients with bipolar disorder compared to patients with unipolar depressive disorder in a naturalistic cohort setting. Results show that MBCT seems to be a feasible and acceptable intervention for patients with bipolar disorder. There were significant less patients with bipolar disorder who decided to start MBCT after intake compared to patients with unipolar depressive disorder. No differences in dropout between groups were observed. Furthermore, we found significant effects in patients with bipolar and unipolar depressive disorder following MBCT on all outcome measures, showing reductions in depressive symptoms and worry, and improvements in well-being, mindfulness skills, and self-compassion. In addition, we did not find any indication that MBCT was less effective in patients with bipolar compared to patients with unipolar depressive disorder. These results lend support to the notion that MBCT, when delivered in heterogeneous patient groups, might be as helpful in patients with bipolar as those with unipolar depressive disorder.

The finding that MBCT seems to be a feasible and acceptable intervention for patients with bipolar disorder is consistent with an earlier feasibility trial conducted by Weber et al. (2010). In contrast to earlier studies on the feasibility of MBCT for patients with bipolar disorder, the present study showed a much lower dropout rate. Results showed a dropout of only 6.6%, compared to dropout rates of 21–34% in the studies of Weber et al. (2010), Williams et al. (2008), and Perich et al. (2013). The low drop-out rate found in the present study as compared to other scientific studies might be explained by the naturalistic nature of the present study. During intake, the motivation of patients is extensively assessed. Patients are only eligible to participate in MBCT when they indicate to be able to attend at least six out of eight sessions, and are motivated to do their homework. Furthermore, when other evidence-based interventions seem more suitable, e.g. trauma therapy, patients are advised not to follow MBCT yet. These considerations might be beneficial for other professionals as well, in order to reduce drop-out in interventions. Furthermore, the present study showed a high completion of measures, with 72% of completers in the present study compared to 67% in the study of Bos et al. (2014).

The findings that MBCT might be as helpful in patients with bipolar as to those with unipolar depressive disorder add to the growing body of evidence of controlled studies, which, although using homogeneous and restricted groups of patients with bipolar disorder, seem to support the possible application of MBCT in bipolar disorder (Deckersbach et al. 2012; Miklowitz et al. 2009; Weber et al. 2010). Furthermore, the results are in line with an earlier study of Williams et al. (2008) where MBCT was found equally effective in reducing depressive symptoms in patients with bipolar and unipolar depressive disorder compared to a waitlist control group. However, the study of Williams et al. (2008) included patients with an explicit history of suicidal ideation or behavior only, which limits the generalizability of the findings to the general population of patients with bipolar and unipolar depressive disorder.

The present study found a non-significant between-groups effect on reduction of worry. Previous studies have indicated that reduction of rumination might be one of the key underlying mechanisms of MBCT (Gu et al. 2015). Our results suggest that MBCT is more effective in reducing levels of worry in patients with bipolar disorder as compared with unipolar depressive disorder. However, clinical diagnosis is conflated with higher levels of rumination. It is known that patients with bipolar disorder show higher levels of rumination than patients with unipolar depressive disorder, regardless of currently experiencing a mood episode (Kim et al. 2012). In our sample, patients with bipolar disorder indeed showed (non-significantly) higher baseline worry than patients with unipolar depressive disorder. Interestingly, studies show that depressive patients with higher baseline levels of rumination, benefit more from MBCT than participants with lower levels of rumination (e.g. Cladder-Micus et al. 2018). Future research will have to elucidate whether the effectiveness of MBCT is best predicted by either clinical diagnosis or baseline levels of rumination. The primary strength of the present study compared to extant literature is the inclusion of a large sample of unipolar depressive patients from a naturalistic cohort. Furthermore, the low dropout rates and high completion of measures are considered to be a strength. However, the results of the present study should be considered in the light of several limitations as well. Firstly, an important limitation is the small sample of patients with bipolar disorder. Since this study uses a naturalistic sample of patients, and MBCT had not been included in the guidelines for bipolar disorder, but had been in the guidelines for unipolar depressive disorder for several years, this is no surprising finding. Secondly, since data of this study was collected as part of ROM, no measures of manic symptom severity were included. However, since we were able to identify reasons for non-completion of MBCT, we are able to show that MBCT did not seem to have precipitated mania in the patients with bipolar disorder who dropped out MBCT in the present study. Thirdly, participants in our study were mainly middle aged, high educated females, which limits the generalizability of our findings. Fourthly, even though the present study shows a similar measurement completion rate in comparison with other clinical trials, the missing data could still result in bias. However, based on the identified reasons for not completing post-measures, we expect this bias to be low. Fifthly, since no follow-up measures were conducted in the present study, we are unaware whether there might have been any adverse effects in patients following MBCT at the long-term. Finally, since this was an uncontrolled study conducted in a naturalistic setting, it did not include a control group.

## Conclusion

This study shows that MBCT seems to be a feasible and acceptable intervention for patients with bipolar disorder. Furthermore, no indications were found that MBCT is less effective in patients with bipolar disorder compared to patients with unipolar depressive disorder. Our findings have some important clinical implications. Reasons for non-completion of MBCT for patients with bipolar disorder were, amongst others, severe depression and suicidality. These possible adverse effects were reported spontaneously in the present study, since this study was not conducted in a controlled research setting. These findings underline the importance of structural monitoring of adverse effects in clinical studies and the need for clear assessment and indication criteria for patients with bipolar disorder to participate in MBCT. A subsequent randomized controlled trial is necessary to establish the effectiveness of MBCT in comparison with TAU, active control conditions or existing evidence-based psychological interventions, such as cognitive behavioral therapy.

## Data Availability

The datasets used and/or analyzed during the current study are available from the corresponding author on reasonable request.

## Abbreviations

BD: bipolar disorder
MBCT: Mindfulness-Based Cognitive Therapy
UDD: unipolar depressive disorder
ROM: Routine Outcome Monitoring
BDI-II: Beck Depression Inventory II
PSWQ: Penn State Worry Questionnaire
FFMQ: Five Facet Mindfulness Questionnaire
SCS: Self-Compassion Scale
OQ-45: Outcome Questionnaire-45
